# Case of Extensively Drug-Resistant *Shigella sonnei* Infection, United States

**DOI:** 10.3201/eid2908.230411

**Published:** 2023-08

**Authors:** Hosoon Choi, Dhammika H. Navarathna, Brennon L. Harston, Munok Hwang, Brandon Corona, Ma Rowen San Juan, Chetan Jinadatha

**Affiliations:** Central Texas Veterans Health Care System, Temple, Texas, USA (H. Choi, D.H. Navarathna, B.L. Harston, M. Hwang, B. Corona, M.R. San Juan, C. Jinadatha);; Texas A&M University, Bryan, Texas, USA (C. Jinadatha)

**Keywords:** extensively drug-resistant Shigella, XDR Shigella, Shigella sonnei, antimicrobial resistance, bacteria, whole-genome sequencing, enteric infections, sexually transmitted infections, men who have sex with men, MSM, United States

## Abstract

We report extensively drug-resistant (XDR) *Shigella sonnei* infection in an immunocompromised patient in Texas, USA. Matrix-assisted laser desorption/ionization time-of-flight mass spectrometry failed to identify XDR *Shigella*, but whole-genome sequencing accurately characterized the strain. First-line antimicrobials are not effective against emerging XDR *Shigella*. Fosfomycin, carbapenems, and tigecycline are potential alternatives.

*Shigella*, the causative agent of shigellosis, can invade human gut mucosa and cause acute bacterial diarrhea. In the United States, antimicrobial resistant *Shigella* infections are frequently associated with men who have sex with men, persons experiencing homelessness, international travelers, immunocompromised persons, and persons living with HIV (*1*). The Infectious Diseases Society of America (https://www.idsociety.org) recommends ciprofloxacin, azithromycin, and ceftriaxone as first-line antimicrobials for shigellosis and trimethoprim/sulfamethoxazole and ampicillin as alternatives. Recently, extensively drug-resistant (XDR) *Shigella* species resistant to all 5 of those recommended agents have rapidly increased. XDR *Shigella* now accounts for 5% of all *Shigella* isolates in the United States (*1*). We describe possible challenges associated with accurately diagnosing a new, emerging strain, XDR *S. sonnei*, because traditional microbiologic tools may fail to identify this pathogen. 

 In January 2023, a man, 33 years of age, sought treatment at an emergency department (ED) for acute onset of loose stools and abdominal pain. The patient reported previous history of recurrent small bowel obstructions because of adhesions from an appendectomy. He first tested positive for HIV in January 2022 and was taking bictegravir/emtricitabine/tenofovir alafenamide. His HIV viral load was undetectable, and CD4 count was 828 cells/μL at the time of admission.

In the ED, we initially treated the patient with 1 dose each of intravenous ciprofloxacin (400 mg) and oral metronidazole (500 mg), along with fluid resuscitation. Upon patient admission, we started him on piperacillin/tazobactam (4.5 mg IV every 6 h [5 doses total]) and oral vancomycin (125 mg 4×/d [4 doses total]). After PCR was negative for *Clostridioides difficile* (Cepheid Xpert C. difficile; https://www.cepheid.com), we discontinued oral vancomycin. Enteric bacterial molecular panel (BD_MAX Extended Enteric Bacterial Panel; Fisher Scientific, https://www.fishersci.com) was positive for *Shigella* spp. On day 2 of his hospital stay, the patient voluntarily discharged against medical advice with 7-day prescriptions for oral doxycycline and oral ciprofloxacin. Antimicrobial and biochemical susceptibility identification results (VITEK Solutions; bioMérieux; https://www.biomerieux.com) were available 1 day after discharge. During follow-up with his primary care physician 2 weeks after being hospitalized, the patient reported that all symptoms of abdominal pain and diarrhea had resolved despite ineffective antimicrobial therapy. 

We isolated a non–lactose fermenter colony forming unit from the cultured fecal sample. Although MALDI-TOF (matrix-assisted laser desorption/ionization-time of flight) mass spectrometry using VITEK MS (bioMérieux) misidentified the isolate as *Escherichia coli*, a VITEK biochemical panel correctly identified the isolate as *S. sonnei*. Using bioMérieux API50 CH strips, we biochemically characterized the isolate, which we classified as *S. sonnei* biotype g (ONPG +, rhamnose –, xylose –) (*2*). Phenotypic antimicrobial susceptibility testing showed the strain was resistant to all 5 antimicrobial drugs recommended for *Shigella* infection (Table). The isolate was resistant to ampicillin/sulbactam, 1st generation cephalosporins, cefuroxime, cefuroxime/axetil, cefpodoxime, ceftazidime, and cefepime, as well as all quinolones and tetracycline. However, that strain of XDR *Shigella* is susceptible to fosfomycin, carbapenems, and tigecycline, which can be used as therapeutic alternatives (Table). In spite of in vitro susceptibilities of the strain to some other antimicrobial drugs—cephalosporins, aminoglycosides, and nitrofurans—they do not penetrate the intestinal mucosa well and so are not recommended for treatment (*1*). 

**Table:** Antimicrobial MICs and putative resistance genes of *Shigella sonnei* strain MB23166 from a case of XDR *S. sonnei* infection, United States*

Antimicrobial	MIC	Interpretation	Putative resistance genes
First-line antimicrobial treatment†			
Ciprofloxacin	≥4	R	qnrB19, *gyrA* (p.S83L)
Ceftriaxone	≥64	R	*bla*_CTX-M-27_
Azithromycin	≥256	R	*mph(A)*
Alternative antimicrobial treatment†			
Ampicillin	≥32	R	*bla*_CTX-M-27_
Trimethoprim/sulfamethoxazole	≥320	R	*sul1*, *sul2*, *dfrA1*, *dfrA17*
Other antimicrobials used for the patient before identification of XDR *Shigella*			
Metronidazole	≥256	R	NA
Piperacillin/tazobactam	64	I	NA
Doxycycline	24	R	*tet(A)*
Potential antimicrobials for XDR *Shigella*			
Fosfomycin	1.5	S	NA
Ertapenem	≤0.5	S	NA
Imipenem	≤0.25	S	NA
Meropenem	≤0.25	S	NA
Tigecycline	≤0.5	S	NA
Mecillinam (pivmecillinam)	0.032	S	NA
Other antimicrobials			
Amoxicillin/clavulanic acid	4	S	NA
Cefotetan	≤4	S‡	NA
Cefoxitin	≤4	S‡	NA
Ceftizoxime	≤1	S‡	NA
Amikacin	4	S‡	NA
Gentamicin	≤1	S‡	NA
Tobramycin	2	S‡	NA
Nitrofurantoin	≤16	S	NA
Aztreonam	4	S	*bla*_CTX-M-27_
Ampicillin/sulbactam	≥32	R	NA
Ticarcillin	≥128	R	*bla*_CTX-M-27_
Piperacillin	≥128	R	*bla*_CTX-M-27_
Cephalothin	≥64	R	NA
Cefazolin	≥64	R	NA
Cefuroxime	≥64	R	NA
Cefuroxime/axetil	≥64	R	NA
Cefpodoxime	≥8	R	NA
Cefotaxime	16	I	*bla*_CTX-M-27_
Ceftazidime	≥64	R	*bla*_CTX-M-27_
Cefepime	≥64	R	*bla*_CTX-M-27_
Nalidixic acid	≥32	R	*gyrA* (p.D87G), *gyrA* (p.S83L)
Levofloxacin	≥8	R	NA
Moxifloxacin	≥8	R	NA
Norfloxacin	≥16	R	NA
Tetracycline	≥16	R	*tet(A)*
Chloramphenicol	16	I	NA

*I, intermediate; NA, not applicable; R, resistant; S, susceptible; XDR, extensively drug-resistant. †According to 2017 Infectious Diseases Society of America guidelines (https://www.idsociety.org). ‡Although susceptible in vitro, not effective clinically for *Shigella* species according to Clinical and Laboratory Standards Institute Performance Standards for Antimicrobial Susceptibility Testing, 32nd edition(https://clsi.org).

Whole-genome sequencing average nucleotide identity analysis determined the isolate was *S. sonnei* (98.56% identity) (*3*). Other closely related species had lower average nucleotide identity values: *S. flexneri* (98.37%), *S. dysenteriae* (97.94%), and *E. coli* NC_011601.1 (96.86%). The closest bacterial genome identified using KmerFinder was *S. sonnei* NZ_CP053751.1 (*4*). The isolate was MLST sequence type 152, the predominant *S. sonnei* isolate (*5*,*6*); cgMLST type was 194163 (*7*).

ResFinder identified putative antimicrobial resistance genes from the genome (Table) (*8*). Extended-spectrum β-lactamase *bla*_CTX-M-27_ was the putative resistance gene against penicillin and cephalosporins. Chromosomal mutation *gyrA* (*p.S83L*) and plasmid-encoded *qnrB19* were the ciprofloxacin-resistant genes of the isolate. *Mph*(*A*) was responsible for azithromycin resistance. *Sul1*, *sul2*, *dfrA1*, and *dfrA17* were the putative resistance genes potentially responsible for trimethoprim/sulfamethoxazole resistance. We found virulence genes using VirulenceFinder (https://cge.cbs.dtu.dk/services/VirulenceFinder) (*9*). *SigA* in the SHI-1 pathogenicity island and *iucC*, *iutA*, *shiA*, and *shiB* in the SHI-2 pathogenicity island were present in the genome (*5*). Other virulence genes in the genome were *anr*, *cia*, *colE7*, *csgA*, *hlyE*, *lpfA*, *nlpI*, *senB*, *sitA*, *terC*, *traT*, *yehA*, *yehB*, *yehC*, and *yehD*. Whole-genome shotgun sequencing and antibiogram results and other information on this isolate are available from the National Center for Biotechnology Information BioSample database (no. SAMN34030354). 

In our study, we found *Shigella sonnei* causing abdominal pain and diarrhea in a patient; MALDI-TOF mass spectrometry initially misidentified the pathogen as *E. coli*, but biochemical testing, confirmed by whole-genome sequencing, correctly identified *S. sonnei.* Clinicians and laboratories should be vigilant for this emerging XDR strain predominantly circulating among HIV-infected MSM (*10*) and aware of its resistance to all commonly recommended empiric and alternative antimicrobial drugs. 
